# GRP78 Level Is Altered in the Brain, but Not in Plasma or Cerebrospinal Fluid in Parkinson’s Disease Patients

**DOI:** 10.3389/fnins.2019.00697

**Published:** 2019-07-05

**Authors:** Jean-Ha Baek, Dejan Mamula, Beata Tingstam, Marcela Pereira, Yachao He, Per Svenningsson

**Affiliations:** Department of Clinical Neuroscience, Karolinska Institute, Stockholm, Sweden

**Keywords:** Parkinson’s disease, unfolded protein response, glucose-regulated protein 78, endoplasmic reticulum stress, neurodegenerative diseases

## Abstract

Accumulation of misfolded proteins results in cellular stress, and is detected by specific sensors in the endoplasmic reticulum, collectively known as the unfolded protein response (UPR). It has been prominently proposed that the UPR is involved in the pathophysiology of Parkinson’s disease (PD). In the present study, the levels of the UPR proteins and mRNA transcripts were quantified in *post mortem* brain tissue from PD patients and matched controls. The level of a key mediator of the UPR pathway, glucose-regulated protein 78 (GRP78), was significantly decreased in temporal cortex and cingulate gyrus, whereas there were no significant changes in the caudate nucleus, prefrontal, or parietal cortex regions. On the other hand, GRP78 mRNA level was significantly increased in caudate nucleus, cingulate gyrus, prefrontal, and parietal cortex regions. GRP78 protein level was also measured in plasma and cerebrospinal fluid, but there were no differences in these levels between PD patients and control subjects. Furthermore, immunofluorescence labeling of the CD4^+^ T cells from PD patients showed that GRP78 protein is found in the cytoplasm. However, GRP78 level in PD patients was not significantly different from control subjects. Unlike the previous Lewy body dementia study, the present investigation reports reduced cortical protein, but increased transcript levels of GPR78 in PD. In summary, these data provide further evidence that GRP78 regulation is dysfunctional in the brains of PD patients.

## Introduction

Parkinson’s disease (PD) is one of the most common neurodegenerative disease affecting 1–2% of the population over 60 years of age (Dorsey and Bloem, 2018). PD is diagnosed based on the presence of bradykinesia, resting tremor, and postural rigidity. PD is characterized by a progressive degeneration of dopaminergic neurons in the substantia nigra pars compacta and deposits of intracellular protein inclusions called Lewy bodies, where aggregates of misfolded α-synuclein (α-syn) are the major components (Spillantini et al., 1997). Although these causative factors for PD have been known for many years and extensive research have been done to halt the disease progression, at present, there are no disease modifying therapies available for PD. Current treatments only restore dopamine neurotransmission and reduce symptoms, but do not stop or slow down the disease progression.

Accumulation of specific misfolded proteins is a salient feature of many neurodegenerative diseases, including PD. The build-up of misfolded proteins gives rise to cellular stress, and is detected by specific sensors in the endoplasmic reticulum (ER). To overcome ER stress, mammalian cells activate a specific signaling pathway in the ER called the unfolded protein response (UPR), which is initiated by the binding of the glucose-regulated protein 78 (GRP78, also known as binding immunoglobulin protein, BiP), an ER chaperone, to misfolded proteins (Harding et al., 1999; Rutkowski and Kaufman, 2004). The UPR consists of three pathways, in which includes ER-resident transmembrane proteins, known as protein kinase RNA-like ER kinase (PERK), inositol-requiring enzyme 1 (IRE1), and activating transcription factor 6 (ATF6). UPR serves as a protective mechanism against the buildup of toxic misfolded proteins, in which first inhibits the protein synthesis, then up-regulate genes that are involved in protein folding or disposal in order to stabilize the disturbed ER homeostasis (Walter and Ron, 2011; Hetz, 2012). The early response of the UPR pathway is activated by PERK (Nakka et al., 2016), which leads to the phosphorylation of the eukaryotic translation initiator factor, eIF2α. This event inhibits general protein synthesis in order to reduce the load on the ER, having an important pro-survival role (Harding et al., 2000; Fernandez et al., 2002). However, under prolonged or chronic stress, phosphorylation of eIF2α increases translation of activating of transcription-4 (ATF4) mRNA, which encodes a transcription factor that induces the expression of pro-apoptotic genes such as the C/EBP-homologous protein (CHOP; also known as DDIT3/GADD153) (Zinszner et al., 1998). Although the initial intention of the UPR activation is to overcome ER stress, if a cell fails to reach proteostasis due to chronic or irreversible ER stress, then the UPR signals to cell death by apoptosis (Tabas and Ron, 2011; Urra et al., 2013).

It has been shown that the UPR is activated in *post mortem* human brains of PD patients (Hoozemans et al., 2007; Selvaraj et al., 2012) as well as in animal and cell models of parkinsonism (Mercado et al., 2016), implying that the neurons are prone to ER stress, and that the UPR may have a role in the degeneration of dopaminergic neuron. Numerous studies have demonstrated that the pathological aggregation/accumulation of α-syn activates the UPR pathway, consequently inducing pro-apoptotic changes (Cooper et al., 2006; Sugeno et al., 2008; Bellucci et al., 2011). Despite the fact that there is a general acceptance of the UPR activation in PD, previous human *post mortem* studies have only focused on demonstrating the presence/existence of UPR activation in PD through semi-quantitative immunohistochemical approach. Therefore, the primary aim of the current study was to accurately quantify the changes in the level of UPR proteins and mRNA transcripts in PD patients in various brain regions by using western blot and quantitative real-time PCR.

The cerebrospinal fluid (CSF) has been extensively investigated as a source of robust biomarkers for neurodegenerative diseases, particularly for Alzheimer’s disease (AD), but also for atypical parkinsonian disorders (Lleo et al., 2015). CSF is the biological fluid closest to the brain as it is not separated from the brain by the blood brain barrier, unlike plasma. However, at the moment, there is no CSF biomarker available to diagnose PD in clinics. The UPR proteins have never been investigated as a potential biomarker for PD. Therefore, the second aim of the present study was to investigate the possibility of the UPR proteins, specifically GRP78 protein, as a novel biomarker for PD.

## Materials and Methods

### Post Mortem Human Brain Tissues

*Post mortem* brain tissue was obtained from the MRC London Neurodegenerative Diseases Brain Bank, United Kingdom. All participants gave informed consent for their tissue to be used in research and the study had ethics approval from the UK National Research Ethics Service (08/H1010/4 and KI IRB) and from the Regional Ethics Review Board of Stockholm (2014/1366-31). The demographic details of the patients and control subjects are shown in Table 1. It is worth to declare that authors did not have any control over the sample collection, and hence, long *post mortem* delays of these brain samples could not be avoided. Biochemical analyses were undertaken on five different brain regions: caudate nucleus (*n* = 36), prefrontal cortex (*n* = 40), temporal cortex (*n* = 41), anterior cingulate gyrus (*n* = 38), and parietal cortex (*n* = 39). Temporal cortex tissues were not available for RNA analysis due to technical reasons. Caudate nucleus was selected for its involvement in motor function in PD; prefrontal cortex was selected for its proposed role in executive function and cognition; cingulate gyrus was selected for the early development of pathology encountered in this region, while parietal cortex was selected because of its pathological predominance in AD as opposed to PD; temporal cortex was chosen due to its suggested role in auditory processing and language.

**Table 1:** Demographical and clinical characteristics of subjects in this study.

Post mortem brain						
**Group**	**Gender (M/F) (%)**		**Age at death**		**Post mortem delay (h)**	

**Caudate nucleus**						
Control (*n* = 18)	50/50		72.1 ± 2.6		31.2 ± 4.7	
PD (*n* = 18)	50/50		75.2 ± 1.8		44.6 ± 5.2	
**Prefrontal cortex**						
Control (*n* = 24)	58/42		80.2 ± 1.5		38.8 ± 4.8	
PD (*n* = 16)	56/44		74.8 ± 2.0		44.8 ± 5.8	
**Temporal cortex**					
Control (*n* = 23)	61/39		80.3 ± 1.6		37.8 ± 4.9	
PD (*n* = 18)	50/50		75.2 ± 1.8		44.6 ± 5.2	
**Cingulate gyrus**					
Control (*n* = 22)	64/36		80.4 ± 1.7		38.5 ± 5.0	
PD (*n* = 16)	50/50		75.5 ± 1.7		45.5 ± 5.8	
**Parietal cortex**					
Control (*n* = 21)	62/38		79.7 ± 1.5		39.0 ± 5.1	
PD (*n* = 18)	50/50		75.2 ± 1.8		44.6 ± 5.2	

**CSF**						

**Group**	**Gender (M/F) (%)**	**Age**	**Disease duration (yrs)**		**H&Y score**	

Control (*n* = 20)	50/50	66.6 ± 2.3	n/a		n/a	
PD (*n* = 19)	47/53	65.1 ± 2.3	5.9 ± 1.3		3 ± 0.2	

**Plasma**						

**Group**	**Gender (M/F) (%)**	**Age**	**Disease duration (years)**	**H&Y score**	**Total UPDRS**	**MoCA**

Control (*n* = 38)	50/50	63.2 ± 2.2	n/a	n/a	n/a	n/a
PD (*n* = 38)	53/47	64.7 ± 1.9	3.1 ± 0.8	2 ± 0.2	38.5 ± 3.6	22.7 ± 1.0

**PBMCs**						

**Group**	**Gender (M/F) (%)**		**Age**			

Control (*n* = 5)	40/60		65.4 ± 3.3			
PD (*n* = 5)	40/60		69.2 ± 3.9			

*PD, Parkinson’s disease; F, female; M, male; n/a, not applicable; H&Y, Hoehn and Yahr; UPDRS, unified Parkinson’s disease rating scale; MoCA, Montreal Cognitive Assessment; PBMCs, peripheral blood mononuclear cells. All measures are presented as mean + SEM, except H&Y score is presented as median ± SEM.*

### Participants for CSF and Plasma Collection

Consents from participants were collected according to the Declaration of Helsinki, which was approved by the regional ethical committees. CSF and plasma samples were collected as described previously (Bjorkhem et al., 2013). All participants fulfilled the clinical diagnostic criteria for PD (Gelb et al., 1999), and PD severity was scored with the Unified Parkinson’s disease rating scale (UPDRS) and Hoehn and Yahr scale. The Montreal Cognitive Assessment (MoCA) scores were also obtained. Control subjects were healthy volunteers or had mild symptoms without any severe neurological diagnosis (e.g., temporary tension headache or sensory symptoms). Control subjects were age- and gender-matched to PD patients (Table 1).

### CSF and Plasma Collection

The standardized lumbar puncture procedure was performed according to the Alzheimer’s Disease Neuroimaging Initiative recommended protocol. CSF was collected into sterile polypropylene tubes, in which the first 2 mL was discarded, and approximately 10–12 mL of CSF from the first portion was collected and gently mixed in order to minimize the gradient influence. Cell counts were measured and samples were centrifuged in the original tube at 1800 × *g* for 10 min at 4°C. Blood was collected in ethylenediaminetetraacetic acid (EDTA) tubes and centrifuged at 800 × *g* for 20 min. Plasma was collected from the top phase of the gradient. Both CSF and plasma were aliquoted in polypropylene tubes, frozen on dry ice and stored at -80°C until use. The maximum time interval from the sample collection until freezing was 30 min.

### Preparation of Tissue Samples for Western Blotting

Western blot samples were prepared as previously described (Baek et al., 2016). Briefly, 100 mg of frozen tissue was taken from each brain region, which was then homogenized in 1 mL of ice cold buffer (pH 7.4) containing 50 mM Tris–HCL, 5 mM ethylene glycol-bis(β-aminoethyl ether)-N,N,N′,N′-tetraacetic acid (EGTA), 10 mM EDTA, “complete protease inhibitor cocktail tablet” (Sigma), phosphatase inhibitor (PhosStop, Sigma), and 2 μg/mL pepstatin A dissolved in ethanol:dimethyl sulfoxide (DMSO) 2:1 (Sigma). Homogenization was performed using disposable pestles (Cat# BELAF199230001, VWR) until the liquid appeared homogenous. Protein concentration of each sample was measured by using BCA Protein Assay Kit (Thermo Fisher Scientific).

### Western Blotting

Twenty micrograms of each sample was loaded on 8% SDS–polyacrylamide gel for protein separation then transferred to nitrocellulose membrane (Immobilon-F, Millipore). After blocking for nonspecific binding, the membranes were incubated with anti-GRP78 (rabbit polyclonal, 1:1000, Cat# ab21685, Abcam), anti-eIF2α (rabbit polyclonal, 1:1000, Cat# 9722, Cell signaling), anti-phosphorylated eIF2α (rabbit polyclonal, 1:1000, Cat# 9721, Cell signaling) primary antibodies followed by IRDye 800CW goat anti-rabbit secondary antibody (1:20,000, Cat# 926-32211, Li-Cor). Bands were detected using an Odyssey infrared fluorescent scanner, and the integral of intensity was quantified using Odyssey infrared imaging system application software version 2.1. β-Actin was chosen as a “house-keeping” protein in order to control for any inconsistency in loading samples. Each membrane was therefore probed for actin (mouse monoclonal, 1:10,000, Cat# A5441-100UL, Sigma–Aldrich) to normalize the level of immunolabeling of the protein-of-interest to actin, so that any potential variations in protein loading could be eliminated.

### RNA Extraction and Quantitative Real-Time PCR (qRT-PCR)

Total RNA was extracted from 30 mg of frozen human brain tissues using RNeasy Plus Mini Kit (Qiagen) according to manufacturer’s protocol. The samples were then measured and evaluated for concentration and purity (260/280 nm ratio) using a Nanodrop (Marshall Scientific). RNA samples were stored at -80°C until use. cDNA was synthesized from 30 ng of total RNA using QuantiTect Reverse Transcription Kit (Qiagen). Levels of human GRP78 (Assay ID: Hs00946084_g1), eIF2α (Assay ID: Hs00187953_m1), and CHOP (Assay ID: Hs00358796_g1) transcripts were measured by qRT-PCR. Briefly, qRT-PCR reactions were prepared in duplicate for each sample with TaqMan assay (Thermo Fisher Scientific) and performed on a CFX96 Real-Time System (BioRad). All reactions were run at 55°C as an annealing temperature and for 40 s for elongation time. Transcript levels were determined by the comparative cycle threshold method and glyceraldehyde-3-phosphate dehydrogenase (GAPDH) (Assay ID Hs02758991_g1) was used as an internal control for normalization.

### Quantification of Circulating GRP78 Protein by ELISA

The levels of GRP78 protein were measured in CSF (neat, i.e., no dilution) and in plasma (1:20 dilution) using the human GRP78 ELISA kit (Enzo Life Sciences) with a detection range of 1.4–4500 ng/mL. The assay was performed according to manufacturer’s instruction.

### Immunofluorescence Labeling of CD4^+^ T Cells

Preparation of peripheral blood mononuclear cells (PBMCs) was performed as previously described (Green et al., 2017). CD4^+^ T cells were isolated by using CD4^+^ T Cell Isolation Kit (Cat# 130-096-533, Miltenyi Biotec) according to manufacturer’s instructions. Purified CD4^+^ T cells were resuspended in proliferation medium (RPMI 1640, glutamine, Pen/Strep, heat inactivated FCS, β-mercaptoethanol, non-essential amino acids, sodium pyruvate) containing Il-2 and Il-7 cytokines. Cells were then seeded in 96-well plates, pre-coated with 5 μg/mL anti-CD3 (clone 2C11), and 1 μg/mL anti-CD28 (clone 37.51) antibodies. The plate was then incubated in a humidified incubator with 5% CO_2_ at 37°C for 7 days. After 7 days of incubation, cells were transferred to 10 μg/mL human ICAM-1-coated μ-slides (Ibidi), and incubated for 45 min at 37°C for adhesion and migration. Cells were then fixed and permeabilized for 20 min using Cytofix/Cytoperm solution (BD Biosciences). Cells were incubated with anti-GRP78 primary antibody (1:50, Cat# ab21685, Abcam), anti-α-syn primary antibody (1:50, Cat# 610787, BD Transduction), anti-calreticulin primary antibody (1:50, Cat# ab22683, Abcam) for overnight at 4°C, followed by goat anti-mouse Alexa Fluor^TM^ 488 secondary antibody (1:500, Thermo Fisher Scientific), goat anti-rabbit Alexa Fluor^TM^ 568 secondary antibody (1:500, Thermo Fisher Scientific) for 1 h at room temperature. All cells were counterstained with DAPI nuclear stain (300 nM, Sigma–Aldrich).

### Confocal Microscopy and Image Analysis

Immunofluorescent images were acquired by ZEISS LSM 880 Airyscan confocal laser scanning microscopy equipped with ZEN2.1 software, using Plan-Apochromat 63×/1.4 Oil DIC M27 63× oil objective. The quantification of the GRP78 protein expression in CD4^+^ T cells was pooled from five independent experiments, in which more than 20 cells were analyzed from each group (control or PD) for every experiment. The analyses were done using ImageJ. The outline of a cell was defined by image threshold, and the total immunofluorescence was measured by maximum projections of Z stack images after background subtraction. The mean fluorescent intensity of PD group was normalized to that of control group.

### Statistical Analysis

Statistical analysis was carried out using GraphPad Prism 5. All descriptive statistics for the variables in the study were reported as means ± standard error of means (SEM), unless otherwise stated. Normality tests were run to assess data distribution. One-way ANOVA or parametric unpaired *t*-test was used for variables with normal distribution, whereas Mann–Whitney non-parametric analysis was used for the distorted distribution. Differences were considered statistically significant with *P* < 0.05.

## Results

### Changes in the Level of GRP78 Protein and the Ratio Between p-eIF2α and Total eIF2α Proteins in Various Regions of the Brain of PD Patients

**FIGURE 1:**
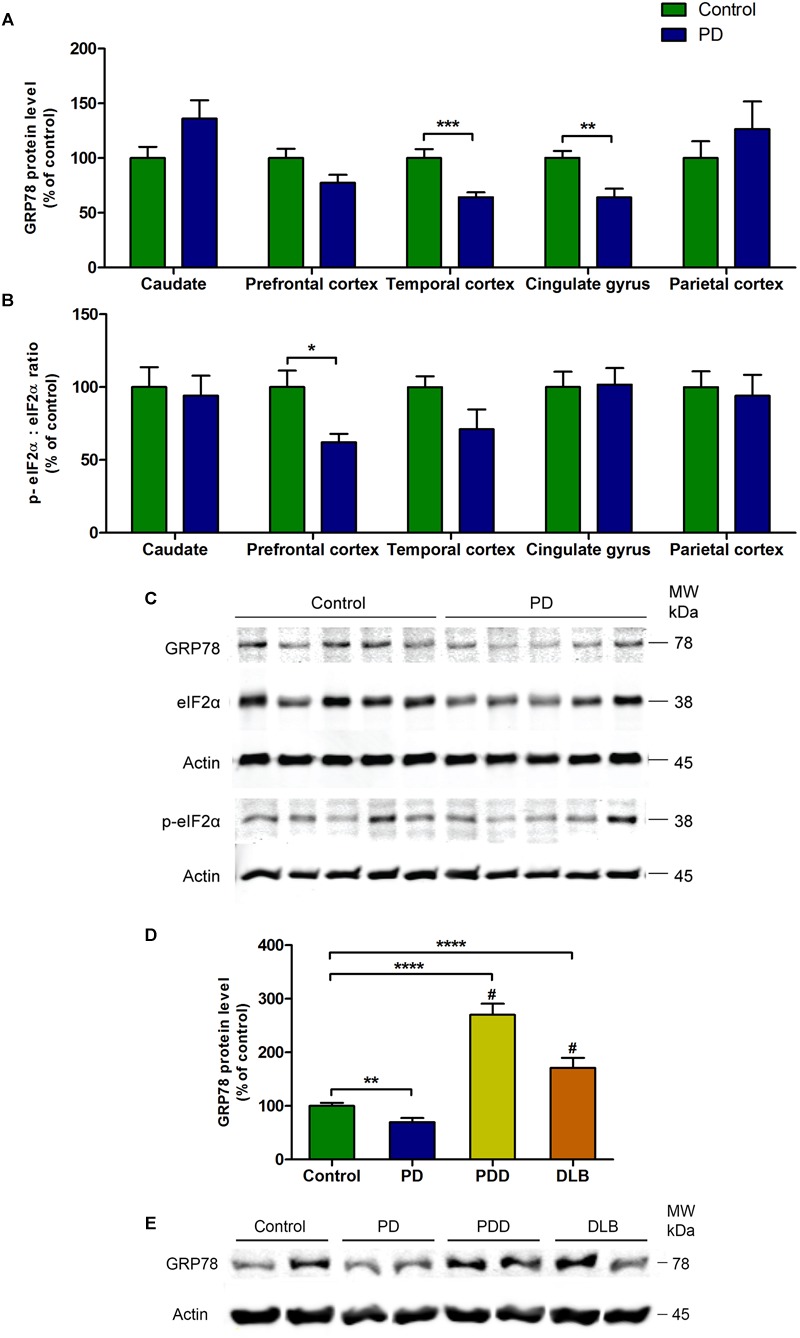
Changes in the level of GRP78 protein and the ratio between p-eIF2α and total eIF2α proteins in various regions of the brain from PD patients and control subjects. **(A)** The level of GRP78 protein was significantly decreased in temporal cortex and cingulate gyrus regions (*P* = 0.0007; *P* = 0.001, respectively). **(B)** The ratio between p-eIF2α and total eIF2α was significantly decreased in prefrontal cortex (*P* = 0.0115). There were no changes in the other regions. **(C)** A representative western blot image showing GRP78, total eIF2α, and p-eIF2α protein expressions in cingulate gyrus region in control and PD subjects. **(D)** The level of GRP78 protein in cingulate gyrus was significantly higher in PD with dementia (PDD) and dementia with Lewy bodies (DLB) patients compared to both control subjects and PD patients (^∗∗^*P* = 0.0023; ^∗∗∗∗^*P* < 0.0001 compared to control; ^#^*P* < 0.0001 compared to PD patients). **(E)** Representative western blot image illustrating the expression of GRP78 protein in cingulate gyrus in control, PD, PDD, and DLB subjects.

There was a significant decrease in the level of GRP78 protein in PD patients compared to control subjects in temporal cortex (*P* = 0.0007) and cingulate gyrus (*P* = 0.001, Figure 1A). Similar pattern was observed in the prefrontal cortex, in which the difference was very close to statistical significance (*P* = 0.0663, Figure 1A). In caudate and parietal cortex regions, there was an increasing trend in the level of GRP78 protein in PD patients compared to control subjects, though statistically insignificant (Figure 1A).

The ratio between phosphorylated-eIF2α (p-eIF2α) and total eIF2α proteins was significantly decreased in PD patients compared to control subjects in prefrontal cortex (*P* = 0.0115, Figure 1B). This decrease was due to a significant increase in the level of total eIF2α protein (*P* = 0.002) in PD patients, while the p-eIF2α protein level remained unchanged (Table 2). In the temporal cortex, p-eIF2α and total eIF2α ratio was decreased almost to a significant level in PD patients compared to control subjects (*P* = 0.0514, Figure 1B). There were no changes in p-eIF2α and total eIF2α ratio in caudate, cingulate gyrus, or parietal cortex (Figure 1B). In order to validate this finding of a general decrease in the levels of GRP78 protein in PD patients, the western blot experiment from Baek et al. (2016) was repeated (Figure 1D,E), in which the authors showed that the levels of GRP78 protein in patients with Parkinson’s disease with dementia (PDD) and dementia with Lewy bodies (DLB) were significantly higher compared to control subjects and AD patients in the cingulate gyrus. As expected, the results were consistent with the results of Baek et al. (2016), in which the level of GRP78 protein in cingulate gyrus was significantly higher in PDD and DLB patients compared to control subjects (^∗∗∗∗^*P* < 0.0001 for both PDD and DLB) and also to PD patients (^#^*P* < 0.0001 for both PDD and DLB) (Figure 1D,E).

**Table 2:** Summary of changes in the UPR proteins in PD patients compared to control subjects.

Brain regions	UPR proteins			
	GRP78	p-eIF2α	eIF2α	p-eIF2α:eIF2α
Caudate	ns	ns	ns	ns
Prefrontal cortex	ns	ns	**↑** *P* = 0.002	**↓** *P* = 0.0115
Temporal cortex	**↓** *P* = 0.0007	**↓** *P* = 0.0014	**↓** *P* = 0.0008	ns
Cingulate gyrus	**↓** *P* = 0.001	ns	ns	ns
Parietal cortex	ns	**↓** *P* = 0.0006	**↓** *P* = 0.0145	ns

*Upward arrow: An increase in expression. Downward arrow: A decrease in expression. ns: Not significant.*

### Changes in the mRNA Level of *Grp78, eif2α*, and *Chop* in Various Regions of the Brain of PD Patients

**FIGURE 2:**
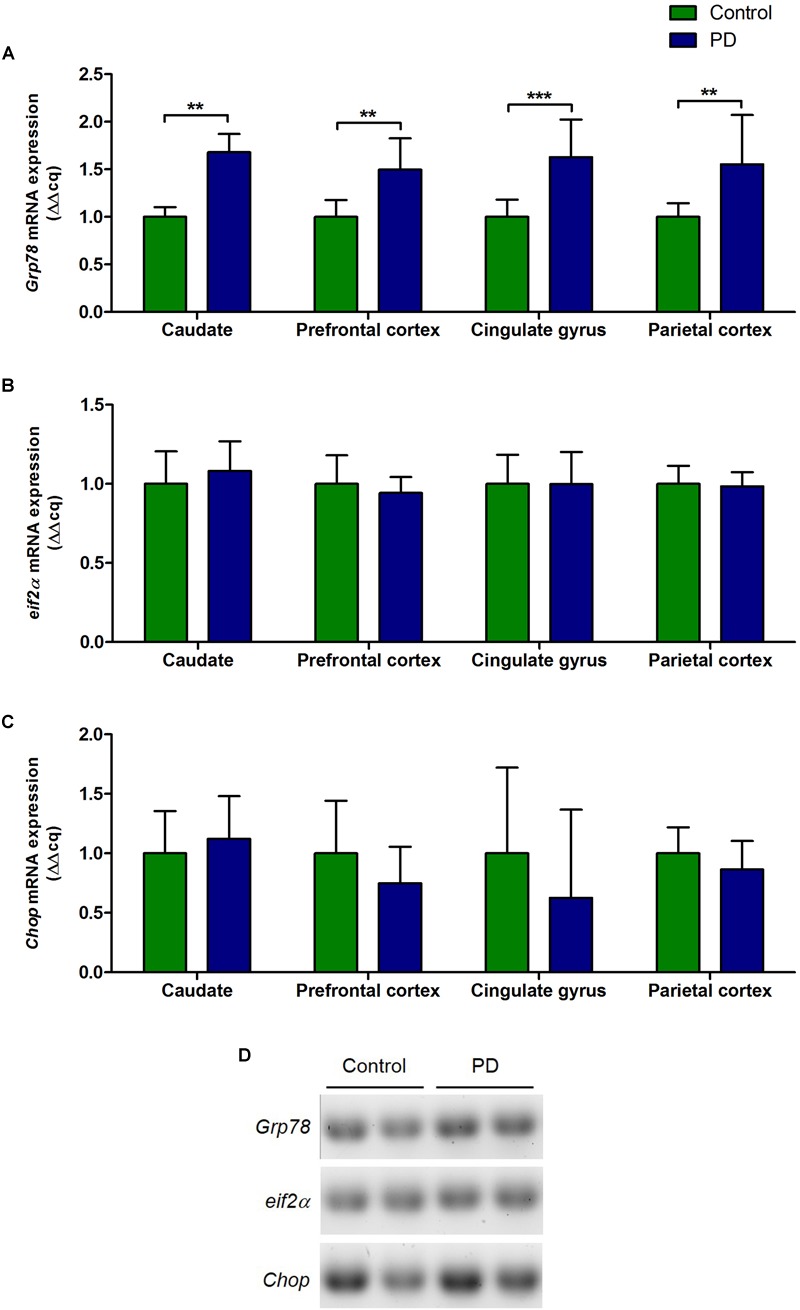
Changes in the level of *Grp78, eif2α*, and *Chop* mRNA expression in various regions of the brain from PD patients and control subjects. **(A)** The level of *Grp78* mRNA in PD patients was significantly increased in all regions of the brain compared to control subjects (*P* = 0.0015, 0.0024, 0.0007, 0.0047 respect to brain regions). **(B,C)** There were no changes in the levels of *eif2α* and *Chop* mRNA in PD patients in any of the brain regions. **(D)** Representative gel image showing the expression of *Grp78, eif2α*, and *Chop* mRNA in prefrontal cortex in control subjects and PD patients.

There were significant increase in the levels of *Grp78* mRNA transcripts in all regions of the brain in PD patients compared to control subjects (caudate, *P* = 0.0015; prefrontal cortex, *P* = 0.0025; cingulate gyrus, *P* = 0.0007; parietal cortex, *P* = 0.0047; Figure 2A and Table 3). However, *eif2α* and *Chop* mRNA levels in PD patients were not significantly different to control subjects in any of the brain regions (Figure 2B–D and Table 3).

**Table 3:** Summary of changes in the UPR mRNA in PD patients compared to control subjects.

Brain regions	UPR mRNA		
	GRP78	eIF2α	CHOP
Caudate	**↑** *P* = 0.0015	ns	ns
Prefrontal cortex	**↑** *P* = 0.0025	ns	ns
Cingulate gyrus	**↑** *P* = 0.0007	ns	ns
Parietal cortex	**↑** *P* = 0.0047	ns	ns

*Upward arrow: An increase in expression. Downward arrow: A decrease in expression. ns: Not significant.*

### Circulating GRP78 Protein in Plasma and CSF of PD Patients

**FIGURE 3:**
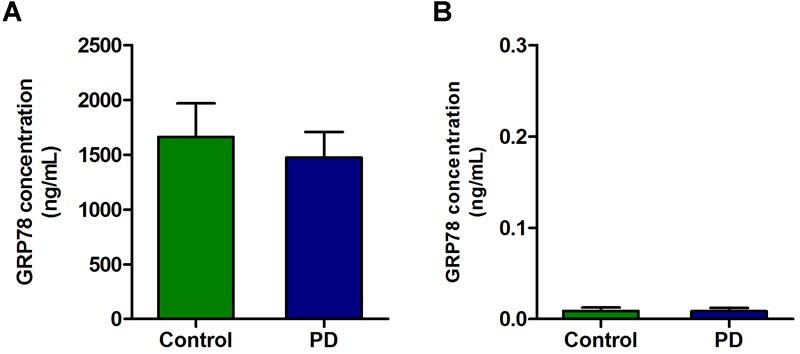
The concentration of GRP78 protein in the plasma and CSF samples of PD patients and control subjects. **(A)** Although there was a modest level of GRP78 protein in plasma of PD patients and control subjects, there was no significant difference between the two groups. **(B)** The GRP78 protein level in CSF was negligible in both PD patients and control subjects.

There was a high concentration of GRP78 protein in the plasma of PD patients as well as in control subjects. Although there was a slight decrease in GRP78 protein level in PD patient compared to control subjects, it was not statistically significant (Figure 3A). In contrast to plasma level, the concentrations of GRP78 protein in CSF of control and PD patients were negligible, and there were no differences between PD patients and control subjects (Figure 3B).

### GRP78 Protein Expression in CD4^+^ T Cells of PD Patients

**FIGURE 4:**
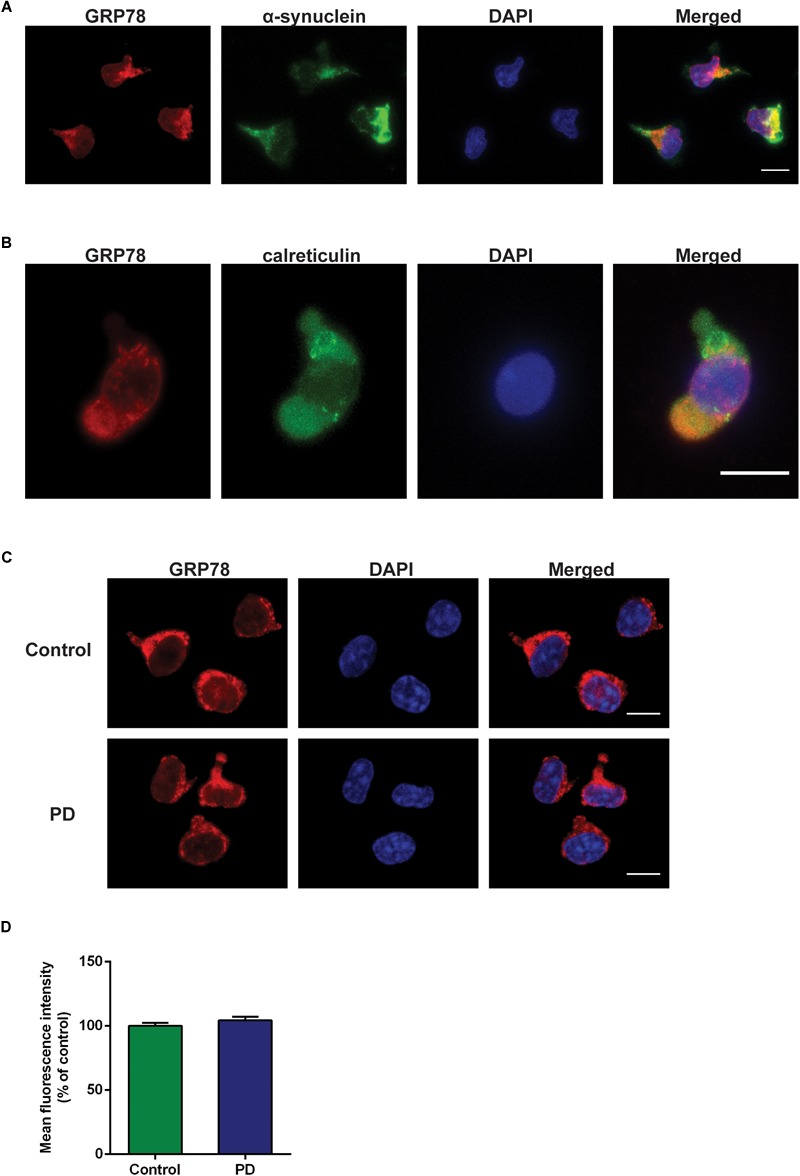
GRP78 protein cellular localization and expression in CD4^+^ T cells derived from control subjects and PD patients. **(A)** Double immunofluorescence staining of GRP78 (red) and α-synuclein (green) in CD4^+^ T cells derived from control subjects. **(B)** Double immunofluorescence staining of GRP78 (red) and calreticulin (green) in CD4^+^ T cells derived from control subjects. **(C)** There was a moderate level of GRP78 protein expression (red) in both control subjects and PD patients. GRP78 was localized in the cytoplasm. Scale bar represents 10 mm. **(D)** The level of GRP78 protein expression in PD patients was not significantly different from control subjects.

Double immunofluorescence staining data with α-syn and calreticulin showed cytoplasmic localization of GRP78 in CD4^+^ T cells (Figure 4A,B). The GRP78 protein was expressed in the cytoplasm of CD4^+^ T cells in both control subjects and in PD patients (Figure 4C). However, the level of expression in PD patients was not significantly different from that of control subjects (Figure 4D).

## Discussion

Growing experimental evidence suggests that UPR is involved in PD; and this is not surprising since the accumulation of misfolded α-syn is a central pathogenic process in PD. Indeed, changes in the expression of GRP78 protein and other UPR activation markers (p-PERK and p-eIF2α) have been observed in the brain of PD patients (Hoozemans et al., 2007; Selvaraj et al., 2012; Baek et al., 2016; Mercado et al., 2018). Nevertheless, there are only a handful of studies examining UPR activation in PD using *post mortem* human brain tissues, and furthermore, almost all of these human studies employ semi-quantitative immunohistochemical method. In the present study, changes in the levels of UPR proteins in various regions of the brain from PD patients were measured by means of quantitative western blot. Surprisingly, among five regions analyzed, there were significant decreases in the level of GRP78 protein in temporal cortex and cingulate gyrus of PD patients compared to control subjects, while there were no changes in caudate, prefrontal, or parietal cortical regions (Figure 1A). Although the observed decrease in the level of GRP78 protein in PD patients is in contrast to previous *post mortem* human studies, this decrease has been observed in other neurodegenerative diseases and during normal aging. For example, in the brain of AD patients, the level of GRP78 protein has been shown to be reduced compared to control subjects (Katayama et al., 1999; Baek et al., 2016) or remained unchanged (Sato et al., 2000). In a mouse model of over-expressing mutated human presenilin-1 gene, the most prevalent mutation found in cases of familial AD, the expression of GRP78 was also found to be decreased (Katayama et al., 1999). Furthermore, Salganik et al. (2015) have shown the loss of GRP78 during normal aging, in which the old rats showed significantly lower levels of GRP78 protein in the nigrostriatal system compared to young animals. In the same study, it was shown that knockdown of GRP78 by specific small interfering RNAs in a rat model of over-expressing α-syn in the substantia nigra aggravated α-syn neurotoxicity in nigral dopamine neurons, which then lead to significantly greater neuronal loss and reduction of striatal dopamine. Moreover, the degree of GRP78 decline was correlated to the severity of neurodegeneration (Salganik et al., 2015).

In the present study, it was shown that changes in the level of GRP78 protein in a particular brain region did not directly correlate with the changes in the levels of downstream proteins such as eIF2α or p-eIF2α (Figure 1B). For instance, in the cingulate gyrus, there was a significant decrease in GRP78 protein level in PD patients compared to control subjects (Figure 1A), but there was no change in the ratio between p-eIF2α and eIF2α levels (Figure 1B). Similar results were found in a rodent study, in which neuronal GRP78 induction in α-syn over-expressing transgenic mice was not accompanied by an increase of p-eIF2α level, suggesting that α-synucleinopathy is linked to abnormal UPR which in turn could trigger cell death (Colla et al., 2012a). Taken together, absence of coherent changes in the level of proteins in the UPR pathway observed in the current study provide additional support for the above hypothesis that PD may be associated with impaired UPR.

Unlike GRP78 protein, the levels of GRP78 mRNA were significantly increased in PD in all areas of the brain analyzed (Figure 2A). However, mRNA levels of eIF2α or CHOP, a pro-apoptotic transcription factor, did not show any significant changes in PD patients compared to control subjects (Figure 2B,C). To explore the possibility whether the increased GRP78 mRNA expression is a compensatory response to the decreased protein level, individual GRP78 mRNA level was correlated to its matching protein level. Nevertheless, no correlation was found (data not shown). The mismatch between the levels of GRP78 mRNA and protein in PD further indicates that the UPR signaling may be dysfunctional, and that PD-related pathology that causes ER stress, likely the accumulation of misfolded α-syn, in some way impair the induction of GRP78 protein, which may also indicate an increase of the vulnerability of neurons to ER stress. Indeed, it was shown that α-syn inhibited processing of ATF6 directly via physical interactions and indirectly by inhibiting ER to Golgi transport of COPII vesicles (Credle et al., 2015). Moreover, the disease-causing mutant α-syn also reduced ER to Golgi trafficking and aggravated ER stress (Thayanidhi et al., 2010; Colla et al., 2012a,b). The phenomenon that decrease in UPR signaling leading to a possible increase in sensitivity to ER stress was demonstrated by Katayama et al. (1999). They showed that inhibition of endogenous IRE1 significantly increased vulnerability to ER stress, and increase in sensitivity to ER stress caused by treatment of an ER stress inducer was reversed by the expression of recombinant GRP78. The results from Katayama et al. (1999), together with the current study, suggest that activation of UPR signaling is important for protective effects against the ER stress, and that the reduction of GRP78 protein level may cause vulnerability to ER stress in PD, which may then potentiate disease progression.

It has been hypothesized that with aging and/or disease progression, soluble α-syn monomers that are present in the ER form insoluble α-syn oligomers/aggregates and attribute to chronic ER stress and neurodegeneration (Colla et al., 2012a). Recently, a new ER stress rat model using intranigral injection of a well-known ER stress inducer, tunicamycin, was employed to investigate whether ER stress is able to induce PD features (Coppola-Segovia et al., 2017). It was shown that ER stress not only induced locomotor impairment and dopaminergic neuronal loss, but also substantial α-syn oligomerization in substantia nigra pars compacta, astroglial activation, and increased expression of ER stress markers (Coppola-Segovia et al., 2017). These results reinforce the notion that ER stress, hence the UPR, could be an important contributor to the pathophysiology of PD. Nevertheless, there are still not enough evidence to decipher the fact whether the activation of the UPR is a cause or consequence of neurodegeneration observed in PD. Furthermore, how and what controls the changeover switch of the UPR between neuroprotection and neurotoxicity remains largely obscure.

While the present study confirms that there is a turbulence in the UPR in different areas of the brain of PD patients, due to technical limitations with reagents, it was difficult to efficiently evaluate the state of other UPR reporters such as XBP1 and PERK. Since induction of the ER chaperones and the XBP1 cleavage can occur independent of UPR mechanisms (Kim et al., 2008), lack of GRP78 and/or p-eIF2α inductions in PD patients observed in the present study may reflect activation of processes other than UPR. Moreover, to further validate the UPR changes observed in the present study, changes in the level of other ER resident proteins, such as GRP94, calnexin, and protein disulfide isomerase, should also be examined in the future.

Given that the UPR is a homeostatic stress response, it means that it is greatly controlled by positive and negative feedback loops. The interaction between the three signaling pathways of the UPR proposes that the alteration of one pathway will affect signaling of the other two pathways also. For example, inhibition of one pathway may in fact increase signaling through one of the other pathways. This phenomenon was demonstrated by Harding et al. (2001) where deletion of PERK resulted in an increased activity of IRE1α. Therefore, additional studies investigating the changes in the proteins of the other two arms of the UPR pathway, that is, IRE1α and ATF6, are needed to fully understand the relationship between different arms of the UPR pathway in PD, and how each arm of the UPR pathway is involved in the neurodegenerative process in PD.

Diagnosis of PD remains tricky and misdiagnosis rate with vascular or atypical parkinsonian disorders reaches up to 20–30% (Rajput and Rajput, 2014). At present, the assessment of clinical motor symptoms underlies the diagnosis of PD. The absence of a reliable biomarker with high sensitivity and specificity has significantly hindered the validation of potential therapies. One of the novel results of this study was the measurement of the GRP78 protein in plasma and CSF samples of PD patients as to discover a potential biomarker for PD. Although GRP78 protein has been detected in the plasma of endometrial cancer and obese patients (Ciortea et al., 2016; Khadir et al., 2016), it has never been measured in PD patients. In the plasma from control subjects and PD patients, high levels of GRP78 protein were detected, though its level in PD patients was not significantly different to control subjects (Figure 3A). To the best of authors’ knowledge, the present study is first to evaluate the presence of GRP78 protein in CSF of PD patients. Nevertheless, unlike plasma, GRP78 protein in CSF was almost undetectable in both control subjects and PD patients (Figure 3B). A possible reason for the undetectable level of GRP78 in CSF could be that “whole” GRP78 protein is too large to be secreted in CSF. Therefore, it would be interesting to investigate whether fragments of GRP78 protein could be detected in CSF. Although the current study did not provide a clear evidence that GRP78 could serve as a possible biomarker for PD, further studies are required in order to investigate whether other UPR proteins and/or ER stress-related proteins have potential to be novel biomarkers for PD. A relatively high level of GRP78 protein in plasma from both control subjects and PD patients (Figure 3A) led us to further understand the implication(s) of this result in the disease state. Therefore, we investigated the level of GRP78 protein expression in PBMCs, specifically in CD4^+^ T cells derived from control subjects and PD patients. However, when we compared the expression level of GRP78 in PD patients vs. control subjects, we found no difference (Figure 4C,D). Delpino and Castelli (2002) showed that extracellular GRP78 are mostly derived from an active release from living cells and are not solely due to the protein leakage from dead cells. Recent studies have also demonstrated that GRP78 release is increased in cancer, obesity, or upon ER stress (Khadir et al., 2016; Steiner et al., 2017). Taken together, it may be hypothesized that the high level of GRP78 protein observed in plasma of PD patients could be due to circulating PBMCs releasing GRP78 into the extracellular domain. However, since the level of GRP78 protein in plasma in PD patients was not different from control subjects, further investigation is inevitable to determine the difference in the state of GRP78 protein between control subjects and PD patients.

## Conclusion

In conclusion, the present study showed that there are changes in the level of UPR proteins and mRNAs, particularly GRP78, in various regions of the brain of PD patients (Figure 1, 2). However, while there were central changes, there were no peripheral changes, as observed in the levels of GRP78 protein in CSF, plasma, and in immune cells (Figure 3, 4). Based on these results, one can cautiously postulate that UPR changes may be limited to the site of neurodegeneration, and not influenced elsewhere. In other words, it may be that the UPR in PD is quite a specific response in terms of location of action rather than a generic reaction to ER stress or progression of PD. This highlights an attractive opportunity to explore the UPR as a novel therapeutic target for PD with negligible peripheral side effects.

## Data Availability

All datasets generated for this study are included in the manuscript and/or the Supplementary Files.

## Ethics Statement

This study was carried out in accordance with the recommendations of the Alzheimer’s Disease Neuroimaging Initiative recommended protocol with written informed consent from all subjects. All subjects gave written informed consent in accordance with the Declaration of Helsinki. The protocol was approved by the UK National Research Ethics Service and by the Regional Ethics Review Board of Stockholm.

## Author Contributions

J-HB and PS conceived and designed the experiments. J-HB, BT, MP, DM, and YH performed the experiments. J-HB, BT, MP, and DM analyzed the data. J-HB wrote the manuscript. All authors have read and approved the final manuscript.

## Conflict of Interest Statement

The authors declare that the research was conducted in the absence of any commercial or financial relationships that could be construed as a potential conflict of interest.
